# Older People's Quality of Life (OPQOL) scores and adverse health outcomes at a one-year follow-up. A prospective cohort study on older outpatients living in the community in Italy

**DOI:** 10.1186/1477-7525-9-72

**Published:** 2011-09-05

**Authors:** Claudio Bilotta, Ann Bowling, Paola Nicolini, Alessandra Casè, Gloria Pina, Silvia Veronica Rossi, Carlo Vergani

**Affiliations:** 1 Department of Internal Medicine, University of Milan, Milan, Italy; 2 Geriatric Medicine Outpatient Service, Department of Urban Outpatient Services, Istituti Clinici di Perfezionamento Hospital, Milan, Italy; 3 Faculty of Health and Social Care, St George's Hospital, University of London and Kingston University, London, UK; 4 Geriatric Medicine Unit, Fondazione IRCCS Cà Granda Ospedale Maggiore Policlinico, Milan, Italy

## Abstract

**Background:**

There is limited knowledge on the ability of a poor quality of life (QOL) and health-related QOL (HRQOL) to predict mortality and other adverse health events, independently of the frailty syndrome and other confounders, in older people living in the community and not selected on the basis of specific chronic conditions. Aim of this study was to evaluate the ability of the overall QOL and of the HRQOL to predict several adverse health outcomes at a one-year follow-up in an older outpatient population living in the community.

**Methods:**

We carried out a prospective cohort study on 210 community-dwelling outpatients aged 65+ (mean age 81.2 yrs) consecutively referred to a geriatric clinic in Milan, Italy. At baseline participants underwent a comprehensive geriatric assessment including evaluation of overall QOL and HRQOL by means of the Older People's Quality of Life (OPQOL) questionnaire. At a one-year follow-up, between June and December 2010, we investigated nursing home placement and death in all 210 participants as well as any fall, any admission to the emergency department (ED), any hospitalisation and greater functional dependence among the subset of subjects still living at home.

**Results:**

One year after the visit 187 subjects were still living at home (89%) while 7 had been placed in a nursing home (3.3%) and 16 had died (7.7%). At multiple logistic regression analyses the lowest score-based quartile of the OPQOL total score at baseline was independently associated with a greater risk of any fall and any ED admission. Also, the lowest score-based quartile of the health-related OPQOL sub-score was associated with a greater risk of any fall as well as of nursing home placement (odds ratio [OR] 10.03, 95% confidence interval [CI] 1.25-80.54, *P *= 0.030) and death (OR 4.23, 95% CI 1.06-16.81, *P *= 0.041). The correlation with the latter two health outcomes was found after correction for age, sex, education, income, living conditions, comorbidity, disability and the frailty syndrome.

**Conclusions:**

In an older outpatient population in Italy the OPQOL total score and its health-related sub-score were independent predictors of several adverse health outcomes at one year. Notably, poor HRQOL predicted both nursing home placement and death even after correction for the frailty syndrome. These findings support and enhance the prognostic relevance of QOL measures.

## Background

In developed countries the rapid ageing of the population has brought to the forefront the well-being of older subjects and emphasised the need to identify individuals at greater risk of adverse health outcomes, such as institutionalisation and death, to whom preventive social and sanitary measures should be targeted. Within the scenario of adverse health outcomes poor quality of life (QOL) may hold a double significance: while it is acknowledged to be per se an adverse health outcome there is also growing evidence that it could be able to predict adverse health outcomes. Indeed in the literature the overall QOL and its specific health-related domain (HRQOL) - as well as other subjective variables conceptually related to the QOL like life satisfaction - have been reported to be predictors of specific adverse health outcomes. Life satisfaction has recently been shown to be an independent predictor of mortality up to 20 years after baseline in a large population study in England [1]. To explain the predictive value of life satisfaction in terms of mortality Bowling and Grundy hypothesized that subjective well-being may act as a buffer, moderating the negative effects of adverse circumstances and facilitating the adaptation to ageing [1]. As far as the prognostic relevance of QOL and HRQOL is concerned, their role as independent predictors of death and clinical complications has been demonstrated mainly in particular populations of older patients, either affected by specific chronic diseases or living in specific settings other than the community. Among the more recent studies we would like to cite those conducted on older people suffering from chronic kidney disease [2], lung cancer [3], metastatic prostate cancer [4], type 2 diabetes [5], ischaemic heart disease [6], heart failure [7], as well as those involving hospitalised older people awaiting residential aged care [8] and residents of veteran homes [9].

The relationship between a poor QOL and adverse health outcomes could be due to the fact that a poor QOL is a marker of underlying conditions at high risk of adverse events, such as polipathology, disability, depression and the frailty syndrome [10-14]. In particular, the latter is a common clinical syndrome in older adults, stemming from a decrease in physiological reserves or from a dysregulation of multiple physiological systems, and although its definition and pathophysiology are still a matter of debate it is recognised to carry an increased risk of poor QOL and adverse health outcomes independently of comorbidity and disability [10,11,15-17].

There are very few studies, all of them recently published, that investigated the correlation between HRQOL and mortality in community-dwelling older people. A poor HRQOL, as assessed by using a proxy measure of broader health status such as the SF-36, was demonstrated to predict mortality among community-dwelling older persons in two studies - one in Taiwan [18] and the other in Spain [19] - but this association was not adjusted for the frailty syndrome [18,19]. An Italian longitudinal study showed that HRQOL, as assessed by the EQ-5D, predicted both mortality and first hospitalisation but, although several covariates were controlled for including the level of physical activity, no adjustment was made for the frailty syndrome [20]. Finally, Masel *et al*. reported that the physical component of HRQOL, as measured by the SF-36, predicted mortality independently of frailty and other confounders in older Mexican Americans, but they did not consider other health outcomes besides death [21].

Thus, somewhat limited information is available on the predictive value of QOL or HRQOL in a sample of community-dwelling older subjects not selected on the basis of a specific disease. Nor are we aware of any study evaluating the prognostic significance of both generic QOL and HRQOL not only on mortality but also on a broader spectrum of adverse events that are common and relevant in older populations, such as falls, functional decline, admission to the emergency department (ED) and nursing home placement. Lastly, to our knowledge, no study based on a community-dwelling older population, except one [21], has considered the frailty syndrome as a potential confounder when adjusting the correlation between QOL measures and adverse health outcomes.

Aim of this study was to evaluate the ability of the overall QOL and of the HRQOL to predict at a one-year follow-up, in an older outpatient population referred to a geriatric medicine clinic in Italy, adverse health outcomes such as falls, greater dependence in the basic activities of daily living (BADLs), ED admission, hospitalisation of at least one day, nursing home placement and death.

## Methods

### Design, setting and participants

This prospective cohort study enrolled at baseline 239 community-dwelling outpatients aged 65+ who consecutively attended a first geriatric visit at the Fondazione Cà Granda Ospedale Maggiore Policlinico in Milan, Italy, from June 15 to November 15 2009. All subjects were referred to this outpatient clinic by their general practitioners and underwent a comprehensive geriatric assessment (CGA), which constitutes a standard procedure of the visit. The main reasons for referral were functional decline, recurrent falls, weight loss, suspected cognitive decline, depression and management of multi-drug therapy. An evaluation of the QOL of the participants was performed by means of the Older People's Quality of Life (OPQOL) questionnaire [22,23], which is described below. Exclusion criteria were: not living in the community, severe cognitive impairment, being unable to fill in the questionnaire properly, refusing to answer all items of the questionnaire. Notably, if an informal caregiver/proxy decision maker accompanied the patient he/she was invited to refrain from influencing the choice of the answer, which had to be made by the older participant him/herself. Further details on exclusion criteria, consent to participation and administration of the questionnaire have been given elsewhere [10]. Signed informed consent to the study was obtained from the older participants or from their caregivers/proxy decision makers in the case of elders suffering from dementia. The study protocol received approval by the hospital's ethics committee. One year after the baseline evaluation each participant or his/her caregiver was called on the phone by an investigator blinded to the baseline data in order to collect information about adverse health outcomes by means of a structured interview (please see below).

### Baseline assessment

All subjects received a CGA which included the main socio-demographic characteristics of the participants, functional and physical status, comorbidity, frailty status and QOL. It was carried out during the visit by a geriatrician and a professional nurse. The data collected by the CGA and considered in this study are summarised herein. The socio-demographic characteristics taken into account were: age, gender, years of schooling, yearly family income and living alone. Subjects were considered to be "living alone" if they were living in their principal place of residence without sharing this residence with any other person. Functional status was assessed by means of the scale for the Basic Activities of Daily Living (BADL) (i.e. transferring, eating, bathing, dressing, toileting, continence) [24]. Comorbidity was assessed by means of the Cumulative Illness Rating Scale morbidity (CIRS-m) scale [25] and by considering diagnoses of dementia and depression, which were made according to the criteria of the Diagnostic and Statistical Manual of Mental Disorders fourth edition text revision (DSM-IV-TR) [26].

As far as the diagnosis of frailty is concerned, over the last few years different criteria have been proposed for this syndrome, with those by Fried *et al*. [16] receiving greater consensus [15]. In our study the frailty status of the participants was evaluated according to the recent Study of Osteoporotic Fractures (SOF) criteria, which are regarded to be just as effective as the frailty criteria of Fried *et al*. in predicting adverse health outcomes but are easier to apply [27-29]. Indeed these criteria for the frailty syndrome have been recently found to predict several adverse health outcomes in an older population referred to the same geriatric service in Italy [30]. The SOF index is composed of three items: 1) intentional or unintentional weight loss > 5% in the past year, 2) inability to rise from a chair five consecutive times without using the arms, 3) self-perceived reduced energy level as described by a negative answer to the question "*do you feel full of energy?*". Subjects are considered "frail" if at least two of the three criteria are fulfilled, "pre-frail" if only one criterion is present and "robust" if none of the criteria are present. We also considered the occurrence of specific life events in the year prior to the visit, such as any fall and any admission to the emergency department (ED).

The QOL of the participants was evaluated by means of the OPQOL questionnaire, which has been validated in a multiethnic community-dwelling older population in England [22,23]. Cronbach's alpha coefficient for the Italian outpatient population enrolled in this study was found to be 0.78, i.e. above the 0.70 threshold of acceptability for internal consistency. Moreover, this questionnaire was recently shown not only to have excellent applicability to cognitively normal subjects but also to be applicable to people suffering from mild or moderate dementia in two studies addressing the association of QOL with both frailty status and living status in an older population referred to the same geriatric service in Italy [10,31]. The OPQOL questionnaire consists of 35 statements with the participant being asked to indicate the extent to which he/she agrees with every single statement by choosing one of five possible options among "strongly disagree", "disagree", "neither agree nor disagree", "agree" and "strongly agree". Each of the five possible answers is given a score of 1 to 5 so that higher scores indicate a better QOL. Thus the total score ranges from 35 (the worst possible QOL) to 175 (the best possible QOL). The 35 statements of the questionnaire consider the following aspects of QOL: life overall, health (score range 4-20), social relationships and participation, independence, control over life and freedom, home and neighbourhood, psychological and emotional well-being, financial circumstances, leisure, activities and religion.

### One-year follow-up

At a one year follow-up each participant or his/her caregiver (in the case of subjects suffering from dementia) was administered a structured interview on the phone by an investigator blinded to the baseline data. The adverse health outcomes considered were: any fall, any admission to the emergency department (ED), any hospitalisation (defined as a hospital stay of at least one day) and death occurring during the year after the baseline visit as well as nursing home placement and greater dependence in the BADLs at the time the phone call was made. The latter was investigated by using the BADL scale and was defined as any decline in the BADL score at follow-up as compared to baseline. If the older participant or his/her caregiver was not reached by the first phone call, we made a maximum of four further calls, one week apart. The follow-up therefore spanned a period of six months, from June 15 to December 15 2010.

### Statistical analyses and sample size calculations

In order to reject the null-hypothesis that a poor overall QOL as well as a poor HRQOL at baseline assessment were not associated with the occurrence of any of the above-mentioned adverse health outcomes at a one-year follow-up, we assumed a poor QOL and a poor HRQOL to coincide with the lowest score-based quartiles of the OPQOL total score and the health-related OPQOL sub-score respectively. For each health outcome, comparisons between subjects scoring in the lowest quartiles of these indices and the rest of the sample were performed by means of the chi-squared test or Fisher's exact test. Furthermore, univariate logistic regression analyses were conducted, all of them assuming the specific adverse health outcome as dependent variable and the lowest score-based quartile of the OPQOL total score or health sub-score (i.e. lowest quartile *vs *rest) as the independent variable.

For those adverse health outcomes which were associated with a poor overall QOL or a poor HRQOL at univariate analyses, multiple logistic regression analyses were then performed. All multivariate models were adjusted for age, sex, comorbidity according to the CIRS m score (highest score-based quartile *vs *rest), diagnoses of dementia and depression, socioeconomic characteristics such as years of education (none or no more than 5 years *vs *more than 5 years), yearly income (no more than 10,000 euros *vs *more than 10,000 euros) and living alone. We chose 10,000 euros as the cut-off in yearly income because it is very close to the relative poverty threshold in Italy in 2009 [32]. Also, different adjustments were made to the multivariate models in order to take into account a predisposition to the specific adverse health outcome considered. When death and nursing home placement were taken as dependent variables, corrections were made for those conditions which are well known to be independently related to a greater risk of institutionalisation and death, namely severe dependence in the BADLs (lowest quartile of the BADL score *vs *rest) [33-35] and frailty syndrome diagnosed according to the SOF criteria [27-30]. In particular, we focused on dependence in the BADLs since the BADL index captures disability at a more severe stage of the disabling process than does the IADL index, which considers more complex skills like using the telephone, shopping, preparing meals, housekeeping, doing laundry, taking medications, managing transportation and handling money [36]. When any fall and any ED admission were taken as dependent variables, corrections were made for the occurrence of these events in the year prior to the baseline visit since they could reflect underlying predisposing conditions and thus have a confounding effect on the relationship investigated (please see the Discussion section). In order to justify the entry of the variables in the multivariate models, multi-collinearity was assessed by using the correlation matrices in the multivariable analyses output. They showed there were no correlations greater than 0.58 between variables, indicating there was no multi-collinearity at a basic level (corresponding to correlations greater than 0.8) [37].

As far as sample size calculations were concerned, at baseline we had found a 40% prevalence of any fall in the previous year in subjects within the lowest score-based *tertile *of the OPQOL total score [10]. Thus we assumed a prevalence of any fall at follow-up of about 45-50% in subjects within the lowest *quartile *of the OPQOL score. We also estimated a prevalence of missing cases of about 10-15%. It was therefore calculated that with a sample of 239 participants at baseline and about 200 subjects enrolled at a one-year follow-up the study would have obtained an almost 80% statistical power at a 5% alpha level to detect a difference in the absolute risk of any fall of about 20% between subjects within the lowest quartile of the OPQOL score and the rest of the sample.

## Results

**Figure 1:**
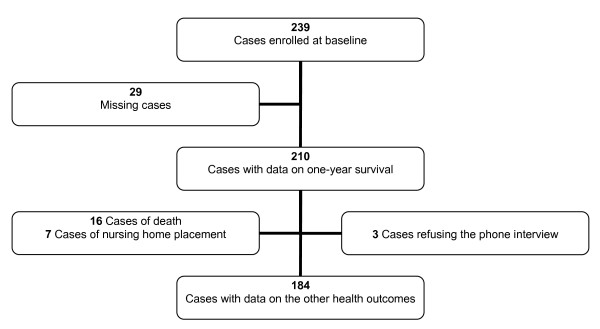
**Enrolment of study participants and disposition of cases at a one-year follow-up**.

Out of the 239 participants enrolled at baseline, 29 were lost to the one-year follow-up: these missing cases were those in which either the patient or his/her caregiver could not be contacted on the phone. Among the remaining 210 participants, 3 patients answered the phone but refused to be interviewed; they nonetheless provided confirmation of their currently living at home so that data on survival and living arrangements one year after the baseline visit were available for all (Figure 1). The main characteristics of the participants at the baseline evaluation are summarised in Table 1. One hundred and eighty-seven subjects were still living at home (89%) while 7 had been placed in a nursing home (3.3%) and 16 had died (7.7%). Data concerning the other adverse health outcomes (i.e. any fall, greater dependence in the BADLs, any ED admission, any hospitalisation) were available for 184 participants, after excluding those participants who had died and had been placed in a nursing home as well as the 3 patients who were still living at home but refused to be interviewed (Figure 1). During the year after the baseline visit, out of these 184 participants 73 subjects (40%) experienced at least one fall, 72 (39%) developed a greater dependence in the BADLs, 61 (33%) had at least one admission to the ED and 46 (25%) at least one hospitalisation.

**Table 1:** Main baseline characteristics of the participants (*n *= 210).

Variables	Percentage (*n*)	Mean (SD)	Lowest quartile	Highest quartile
Age (years)		81.2 (6.5)		86 +
Sex: female	69 (144)			
Education less than or equal to 5 years	36 (75)			
Yearly income < 10,000 euros	17 (35)			
Living alone	45 (94)			
BADL score^a^		4.4 (1.7)	0 - 3	
Any fall in the previous year	31 (66)			
Any ED admission in the previous year	35 (74)			
Being frail (SOF criteria)	31 (65)			
CIRS m score^b^		4.2 (1.8)		5 +
Dementia	28 (58)			
Depression	52 (110)			
OPQOL total score^c^		116.2 (15.4)	35 - 106	
OPQOL health sub-score^d^		10.5 (3.4)	4 - 8	

Notes: SD = standard deviation; ED = emergency department; SOF = Study of Osteoporotic Fractures.

a) Basic Activities of Daily Living. Score range 0 - 6. Lower scores indicate greater dependence.

b) Cumulative Illness Rating Scale morbidity. Scores 0-13. Higher scores indicate greater morbidity.

c) Older People's Quality of Life questionnaire. Score range 35-175. Lower scores indicate worse quality of life.

d) Score range 4-20. Lower scores indicate worse health-related quality of life.

At unadjusted analyses the lowest score-based quartile of the OPQOL total score was associated with a greater risk of any fall (57% [27 out of 47] *vs *34% [46 out of 137], *P *= 0.004) and any ED admission (49% *vs *28%, *P *= 0.008), whereas the lowest score-based quartile of the health-related OPQOL sub-score was associated with a greater risk of any fall (55% *vs *33%, *P *= 0.007), nursing home placement (7% [5 out of 68] *vs *1% [2 out of 142], *P *= 0.037 at Fisher's exact test) and death (13% *vs *5%, *P *= 0.049 at Fisher's exact test) at a one-year follow-up (please see also Table 2 for univariate logistic regression analyses).

**Table 2:** OPQOL total and health-related scores and adverse health outcomes at univariate analyses.

Adverse health outcomes	*N*	Odds Ratio (95% CI)	*P*
OPQOL total score (lowest quartile *vs *rest)			
**Any fall**	184	2.67 (1.36 - 5.26)	0.005
Greater dependence in the BADLs	184	1.36 (0.70 - 2.67)	0.367
**Any ED admission**	184	2.50 (1.26 - 4.95)	0.009
Any hospitalisation	184	1.84 (0.89 - 3.81)	0.100
Nursing home placement	210	2.02 (0.44 - 9.31)	0.368
Death	210	2.18 (0.77 - 6.16)	0.141
OPQOL health-related sub-score (lowest quartile *vs *rest)			
**Any fall**	184	2.40 (1.26 - 4.57)	0.008
Greater dependence in the BADLs	184	0.85 (0.44 - 1.63)	0.616
Any ED admission	184	1.23 (0.63 - 2.38)	0.546
Any hospitalisation	184	1.35 (0.67 - 2.76)	0.404
**Nursing home placement**	210	5.56 (1.05 - 29.41)	0.044
**Death**	210	2.94 (1.05 - 8.27)	0.041

Notes: **Bold variables **are significant at p < 0.05; OPQOL = Older People's Quality of Life questionnaire; CI = confidence interval; BADL = basic activities of daily living; ED = emergency department.

At multiple logistic regression analyses, the lowest score-based quartile of the OPQOL total score (i.e. a score between 35 and 106 out of 175) at baseline was independently associated with a greater risk of any fall and any ED admission (Table 3). The lowest score-based quartile of the health-related OPQOL sub-score (i.e. a score between 4 and 8 out of 20) at baseline was associated with a greater risk of any fall and also with a greater risk of nursing home placement (odds ratio [OR] 10.03, 95% confidence interval [CI] 1.25-80.54, *P *= 0.030) and death (OR 4.23, 95% CI 1.06-16.81, *P *= 0.041). In particular, the correlation between the health-related OPQOL score and the latter two health outcomes was found after correction for age, sex, education, income, living conditions, comorbidity (including CIRS m score, dementia and depression) and the frailty syndrome (Table 4).

**Table 3:** OPQOL score as predictor of any fall and any ED admission at multivariate analyses.

Adverse health outcomes	OPQOL score (lowest quartile *vs *rest) Model adjusted for: **age, sex**, **education, income, living status**, **CIRS m score, dementia, depression**, any fall in the past year (*n *= 184)		OPQOL score (lowest quartile *vs *rest) Model adjusted for: **age, sex**, **education, income, living status**, **CIRS m score, dementia, depression**, any ED admission in the past year (*n *= 184)	
	Odds Ratio (95% CI)	*P*	Odds Ratio (95% CI)	*P*
	
Any fall	2.16 (1.03-4.54)	0.042		
Any ED admission			2.21 (1.05-4.67)	0.037

Notes: OPQOL = Older People's Quality of Life questionnaire; CIRS m = Cumulative Illness Rating Scale morbidity; ED = emergency department; CI = confidence interval.

**Table 4:** Health-related OPQOL sub-score as predictor of any fall, nursing home placement and death at multivariate analyses.

Adverse health outcomes	Health-related OPQOL sub-score (lowest quartile *vs *rest) Model adjusted for: **age, sex**, **education, income, living status**, **CIRS m score, dementia, depression**, **severe dependence in the BADLs**, frailty syndrome (*n *= 210)		Health-related OPQOL sub-score (lowest quartile *vs *rest) Model adjusted for: **age, sex**, **education, income, living status**, **CIRS m score, dementia, depression**, any fall in the past year (*n *= 184)	
	**Odds Ratio (95% CI)**	***P***	**Odds Ratio (95% CI)**	***P***
	
Any fall			2.36 (1.16-4.82)	0.018
Nursing home placement	10.03 (1.25-80.54)	0.030		
Death	4.23 (1.06-16.81)	0.041		

Notes: OPQOL = Older People's Quality of Life questionnaire; CIRS m = Cumulative Illness Rating Scale morbidity; BADL = basic activities of daily living; CI = confidence interval.

## Discussion

This prospective cohort study demonstrated that among community-dwelling older outpatients in Italy poor QOL and HRQOL, as described by the lowest score-based quartiles of the OPQOL total score and health-related OPQOL sub-score respectively, were independent predictors of several adverse health outcomes: falls and ED admissions for overall QOL as well as falls, nursing home placement and death for HRQOL. Our findings lend support to the prognostic value of QOL measures in older people and grant further insight into the association between QOL and adverse health events. As far as the novelty of the study is concerned, some points deserve particular mention. First, to the best of our knowledge, our study provides the first evidence of the predictive value of a poor HRQOL on the occurrence not only of death but also of nursing home placement at one year, after statistical correction for a number of variables including the frailty syndrome. Indeed the latter is an acknowledged predictor of adverse health outcomes, as illustrated in the Background section, and has recently been shown to be the main condition leading community-dwelling older people to death [38]. The choice of the SOF criteria to diagnose frailty is justified by their having been recently validated in large population studies in the U.S. [27-29] and successfully applied to a sample of older subjects attending the same geriatric clinic [30].

Second, the finding that a poor QOL and HRQOL are independently associated with a greater risk of falls at one year is also a novel one. A possible explanation could be that a poor QOL at the baseline visit actually selected a subset of participants who had already experienced falls in the previous year. In fact it is widely recognised that patients who have fallen are at greater risk of further falls [39] and it is equally well known that falls worsen the QOL. This latter effect is mediated by the "fear of falling" syndrome by which older adults who have fallen develop psychological distress and unnecessarily restrict their activity [40]; indeed fall prevention programmes have improved several dimensions of the HRQOL (i.e. physical function, social function, vitality, mental health and environmental domains) in elders living in the community [41]. Yet, the hypothesis of a selection bias does not hold since this association persisted after correction for previous falls at multivariate analysis. An alternative explanation could be that a poor QOL and HRQOL may derive from a number of factors - such as dissatisfaction with one's health, lower social participation or support, negative feelings about the neighbourhood - which reduce the individual's confidence and lead to a constriction of his/her life space. The latter is a measure of spatial mobility, defined as the size of the spatial area people purposely move through in their daily life [42]. Constriction of the life-space is a condition known to decrease physical activity, accelerate physical deconditioning and the decline in physiological reserves [43]: it can be thus speculated that it may increase the risk of falls through a pathophysiological mechanism resembling that of the "fear of falling" syndrome. It can also be supposed that constriction of the life-space contributed to our finding of a correlation between HRQOL and death even after correction for disability and the frailty syndrome: in a population study involving older women, not frail at baseline, it emerged as an independent predictor of both frailty and frailty-free mortality [43]. Of course all hypotheses concerning the relationship between the QOL, life space constriction and adverse health outcomes should be verified by appropriate studies.

Third, another element of novelty of the study resides in the fact that we considered both HRQOL and generic QOL. It is interesting to note that HRQOL and QOL were found to have an impact on different adverse health outcomes. Death and nursing home placement were predicted only by a poor HRQOL, probably because they are mainly due to poor health and poor functional status. ED admissions were instead predicted only by a poor generic QOL. This latter finding suggests that a greater use of the ED by elders is associated with dimensions of the QOL other than the HRQOL, such as dissatisfaction with social support, personal relationships and living environment as well as with a negative perception of one's independence and control over life. In other words, it seems that the subjective distress which makes older people seek help from the ED may be caused not only by physical dysfunction but also by purely social/psychological factors. In keeping with this hypothesis, it has been shown that in older patients discharged from an emergency department in Italy, a multidimensional intervention, based on a CGA performed after discharge, was able to reduce the rate of ED readmissions at a three-month follow-up and was also able to improve not only morale and nutritional status but also generic QOL [44]. It must be emphasised that a poor QOL is associated with several acknowledged predictors of ED admissions such as depressive symptoms, lack of social support, loneliness, larger use of ED visits [45-49]. However, it is noteworthy that in our study this correlation persisted after adjustment for living conditions, depression and previous admissions to the ED.

Finally, some discussion must be devoted to a few methodological issues. When taking falls, ED admissions and hospitalisation as adverse health outcomes we decided for a qualitative rather than a quantitative approach - i.e. we chose to assess the occurrence of any such event in the year after the baseline visit and not the number of events. The latter would in fact have introduced a greater recall bias since it is reasonable to suppose that after a relatively long period of time participants would be able to more accurately report on the absence/presence of adverse events than on the specific number of intervening events. Indeed the reliability of the data so collected is testified by the rate of falls within our sample: we found a 40% prevalence of any fall during one year which appears consistent with figures in the literature - 27% (95% CI 19-36%) according to a review of 18 studies on older community-dwelling subjects [39] - considering the outpatient nature of our population. In fact older subjects referred to a geriatric clinic for health care are likely to be selected for greater comorbidity and risk of adverse events. This same explanation can apply to the high prevalence of frailty, dementia and depression observed in the sample and is supported by the fact that in other recent studies on older outpatients with a disability referred to the same geriatric service the rates of depressive disorders and cognitive impairment were found to be even greater [50,51]. Moreover, it must be noted that frail subjects make larger use of health and community services than subjects who are not frail [52]. Another methodological issue deserving discussion is that we decided to include in the study even subjects suffering from mild or moderate dementia if they were able to understand and reliably answer the OPQOL questionnaire. Such choice was based on the fact that a large proportion of older people can reliably answer questions about their QOL even if they are affected by mild or moderate cognitive deficits. This notion has generally been reported by the literature [53,54] and is consistent with the baseline data of the study, which has specifically shown that the OPQOL questionnaire is applicable to subjects with cognitive impairment [10].

With reference to the limitations of the study, it must be remarked that in the statistical models we found a rather large 95% confidence interval for the odds ratio of nursing home placement and death in relation to the OPQOL health-related sub-score. Although this is certainly not due to multi-collinearity between variables, as previously explained in the Methods, the predictive value of the OPQOL on these two health outcomes needs to be confirmed by further studies conducted on larger samples of community-dwelling older people. Moreover, since the sample analysed consisted of outpatients referred to a geriatric clinic by their general practitioners, our findings cannot be automatically extended to the entire population of older people living at home in Italy. Although we cannot exclude that we might have selected a group of community-dwelling older adults with better social and health assistance, a selection based on economic status can certainly be ruled out since in the specific Italian setting all citizens are granted free access to outpatient services. However, the possible occurrence of a selection bias does not invalidate the clinical relevance of our results and indeed may enhance it. First, the predictive value of the OPQOL score was established in what could be a "best scenario" population. In fact, among the subjects recruited at baseline we lost to follow-up the older and sicker ones who were likely to exhibit greater vulnerability. Moreover - and foremost - all the subjects considered had undergone a CGA and had received individually-tailored therapeutic advice focused on improving their health and QOL, which is the standard approach of geriatric outpatient visits. This highlights the fact that, within the CGA, the administration of the OPQOL questionnaire to evaluate the QOL - particularly in its health-related domain - could better identify those high-risk subjects to whom additional measures should be targeted. Even though specific treatments for frail and vulnerable older patients are yet to be developed and clinically tested [15], and although QOL has seldom been shown to be improved in the very few randomised controlled trials targeting even QOL in frail older people [55,56], our findings underscore the need for research along this line employing also QOL measures such as the OPQOL.

## Conclusions

In an older outpatient population in Italy who had received therapeutic advice based on a CGA, the OPQOL total score and its health-related sub-score were independent predictors of several adverse health outcomes at one year. In particular, poor HRQOL predicted both nursing home placement and death even after correction for severe dependence in the BADLs and frailty syndrome. These findings support the importance of measuring the patients' own perspectives on their lives and enhance the prognostic relevance of QOL measures. Therefore the OPQOL questionnaire could be used, at least in outpatient settings, as a tool to screen older subjects for vulnerability to poor health outcomes and thus better plan appropriate interventions to improve their prognosis.

## Competing interests

The authors declare that they have no competing interests.

## Authors' contributions

CB was responsible for the data, contributed to the literature review, study design, statistical analyses and drafted the manuscript. AB developed the OPQOL questionnaire, contributed to the literature review and revised the manuscript. PN was involved in data collection and revised the manuscript. AC, GP and SVR were involved in data collection. CV was responsible for the data, contributed to the literature review and revised the manuscript. All authors have read and approved the final manuscript.
